# Assessing the impact of perennial groundcover and interseeded annual cover crops on maize yield and drainage water quality

**DOI:** 10.1002/jeq2.70175

**Published:** 2026-04-02

**Authors:** Gabrielle M. Myers‐Bailey, Philip E. Rockson, Daniel S. Andersen, Kenneth J. Moore, Matthew J. Helmers, D. Raj Raman

**Affiliations:** ^1^ Department of Agricultural and Biosystems Engineering Iowa State University Ames Iowa USA; ^2^ Wisconsin Cranberry Research and Education Foundation Black River Falls Wisconsin USA; ^3^ Department of Agronomy Iowa State University Ames Iowa USA

## Abstract

The low adoption of annual cover crops in the United States Corn Belt has motivated research into novel cover cropping systems that mitigate the degrading of water quality due to soil and nutrient loss without compromising corn (*Zea mays* L.) yield. This 3‐year field study compared a Kentucky bluegrass (*Poa pratensis* L.) as a perennial groundcover (PGC), two interseeded annual cover crop systems (standard and wide‐row), and a no‐cover control for their effects on corn yield and subsurface drainage water quality. Each plot was monitored weekly for subsurface drainage flow and nutrient losses using a randomized complete block design with three replicates. The study occurred during a period of below‐average precipitation, which caused strong year‐to‐year and seasonal variations in treatment performance. Treatment effects were most evident in the study's wettest year, when increased rainfall following a dry season caused a summer nitrate flush. During this high‐leaching summer period, all cover crop systems reduced nitrate concentrations compared to the control. When averaged across the entire study, the high‐biomass wide‐row interseeded system reduced flow‐weighted NO_3_‐N concentrations by 22% but reduced grain yield 20%. In contrast, the PGC system reduced NO_3_‐N by 20% with no associated grain yield loss. These findings highlight a critical yield trade‐off for wide‐row annual cover cropping systems and suggest that perennial systems like Kentucky bluegrass PGC are a promising strategy for mitigating early summer nitrate losses.

## Plain Language Summary

Midwest corn farms use underground drainage pipes to help crops grow, but these pipes can carry excess nitrogen into lakes and streams. Too much nitrogen harms aquatic life by fueling algal growth and can make drinking water unsafe. Cover crops, planted between main crops, can help reduce this pollution, but some farmers worry they may reduce corn yields. We investigated the impact of various cover crop methods on water quality and corn yields over 3 years in Iowa. We compared corn with no cover crop to systems using continuous grass between rows and annual cover crops planted between standard or wider rows. Annual cover crops in wide rows grew well and reduced nitrogen loss by 22%, but corn yields dropped by 20%. Continuous grass reduced nitrogen loss by 20% without lowering yield, though drought limited its growth. With the right cover crop and management practices, farms can reduce nitrogen loss while maintaining high yields.

**Abbreviations**
FWA: flow‐weighted average
KBG: Kentucky bluegrass
PGC: perennial groundcover
SR‐ICC: standard row interseeded cover crop
TRP: total reactive phosphorus
WR‐ICC: wide row interseeded cover crop

## 1 INTRODUCTION

The agricultural system of the US Midwest region known as the Corn Belt is characterized by its high productivity of corn (*Zea mays* L.) and soybeans (*Glycine max *(L.) Merr.) grown in monocultures. Among other factors, this productivity is facilitated by the widespread implementation of subsurface (“tile”) drainage (Pavelis, 1987; Skaggs et al., 1994). Subsurface drainage provides a low‐resistance pathway for groundwater to reach surface water and thus lowers the water table and drains saturated soils to make land suitable for agricultural production (Skaggs et al., 1994). Subsurface drainage works to improve crop yields by allowing for earlier planting in wet springs, reducing soil compaction, improving soil aeration, and reducing the risk of crop stress due to excess water (Blann et al., 2009; Mourtzinis et al., 2021). Although subsurface drainage in the region was originally deployed to drain only problematically wet areas of fields, modern systems typically drain entire fields (King et al., 2015).

While drainage can reduce surface runoff and the loss of sediment‐bound pollutants (Istok & Kling, 1983), it provides a low‐resistance pathway for water‐soluble nutrients such as nitrate‐nitrogen (NO_3_‐N) and dissolved phosphorus to flow into surface water, reducing the time for soil chemical and biological processes to mitigate their export (McIsaac & Hu, 2004). These nutrient exports, particularly those of nitrogen and phosphorus, have impacts at local, regional, and national scales (Schmidt et al., 2019; Ward et al., 2018). At the national scale, nitrogen is the causative nutrient in development of the Gulf hypoxic zone, where most of the excess nitrogen loading is attributed to agriculture in the Mississippi‐Atchafalaya River Basin (Goolsby, 2000). As much as 52% of the nitrogen load in the Mississippi‐Atchafalaya River Basin may be attributed to the US state of Iowa alone, where over 50% of the cropland is drained by tile (Jones et al., 2018; USDA NASS, 2019). While surface runoff and erosion are generally seen as the primary mechanisms of phosphorus transport, tile drains can be a significant contributor to surface water phosphorus in agricultural watersheds (King et al., 2015). Phosphorus is a primary cause of freshwater eutrophication, and relatively low concentrations of dissolved P can cause ecosystem harm (Heathwaite & Dils, 2000).

Cover cropping, a practice involving the incorporation of plants beyond the primary cash crop into a crop rotation to extend the duration of (or increase total) soil cover in an annual cropping system, has been promoted as a method for minimizing the negative water quality impacts of the Corn Belt agricultural system (USDA NRCS, 2014). Annual cover crops improve water quality by immobilizing nutrients during fallow periods that could otherwise be lost to runoff or leaching and by protecting the soil from erosion (Christianson et al., 2021; Dougherty et al., 2020; Kaspar et al., 2012; Siller et al., 2016; Strock et al., 2004; Thapa et al., 2018; Waring et al., 2020; Zhu et al., 1989).

Although cover crops have been proven effective in advancing water and soil quality goals, adoption rates are low, with recent estimates finding that just 7% of cropland in the Corn Belt utilizes cover crops (Zhou et al., 2022). Annual cover cropping systems require increased labor and seed inputs at the farm each year, with little or no direct short‐term return on investment and a non‐zero potential to reduce grain yields (Deines et al., 2023; Plastina et al., 2023). Most cover crop systems involve planting the annual cover crop species after the harvest of the cash crop in the fall and terminating the cover crop before planting the next season's cash crop (Oliveira et al., 2019; USDA NRCS, 2019). These management operations can conflict with cash crop management, further reducing farmers’ willingness to adopt the system (Myers & Wilson, 2023; Roesch‐McNally et al., 2018).

Cover cropping systems with alternative management timing may encourage adoption and improve environmental outcomes in the region. Interseeding annual cover crops is a possible solution to the timing concern. In this system, cover crops are planted into growing corn prior to canopy development and die over the winter season, eliminating the cash crop harvest and planting conflicts seen in a typical annual cover crop system (USDA NRCS, 2022). The ability of the cover crop to acquire enough water, nutrients, and solar radiation limits fall growth and the options for cover crop species, so planting cover crops in corn's early vegetative growth stage (typically in late spring) may enhance establishment compared to cover crops planted in the fall, improving the ecosystem benefits and minimally impacting corn yields (Brooker et al., 2020; Rusch et al., 2020; Thapa et al., 2018).

A challenge for the growth of interseeded cover crops is that corn is typically planted in 76 cm rows so that as the corn canopies, the between‐row area is completely shaded. Twin‐row corn with wide‐row spacing, a planting configuration of 152 cm centers with pairs of corn rows planted approximately 20 cm apart, can allow solar radiation to penetrate the lower portions of the corn plant and allow for better use of sunlight for photosynthesis (Nelson, 2014). Additionally, the sunlight in the between‐row space in a wide, twin‐row corn configuration may enhance cover crop establishment and biomass production (Black et al., 2023; Kremer, 2019), improving environmental outcomes.

Another alternative cover cropping strategy is the perennial groundcover (PGC) system, which pairs a complementary (shallow‐rooted, low‐growing) perennial plant species grown with the annual row crop (Moore et al., 2019). In contrast to annual cover crops, PGC systems do not require annual replanting, saving on seed and labor inputs while still providing ecosystem services such as soil erosion prevention, excess nutrient uptake, and soil carbon storage (Schlautman et al., 2021). Studies have shown reduced nutrient losses in a PGC system, but this benefit is often associated with a substantial reduction in corn yield (Qi et al., 2011; Siller et al., 2016), and suppression of the PGC is required to reduce yield risk (Bartel et al., 2020, 2022; Schlautman et al., 2021). Previous research has identified Kentucky bluegrass (*Poa pratensis* L.; KBG) as a viable PGC species, especially when suppressed shortly after corn planting (Bartel et al., 2017, 2022; Chen et al., 2022; Elkins et al., 1979; Wiggans et al., 2012). A PGC approach may be particularly useful in light of climate change‐driven precipitation changes in Iowa, with heavier rain in the spring and early summer observed and predicted to continue (Takle & Gutowski, 2020). The presence of a PGC during these seasons could protect the soil from erosion, reduce nutrient losses, and mitigate the impact of the expansion of tile drainage in response to increased spring precipitation (Olowoyeye & Kaleita, 2024).

In this study, we compare the subsurface drainage water quality and yield impacts of the four following systems: (1) A continuous corn‐KBG strip‐tilled, chemically suppressed PGC system; (2) a standard row width interseeded annual cover crop mixture; (3) a wide, twin‐row corn interseeded annual cover crop mixture; (4) a conventional (non‐cover‐cropped) corn production system. Because of the influence of cover crop growth on nutrient leaching (Christianson et al., 2021), we hypothesize cover crop treatments will modify nutrient concentrations and losses seasonally. Specifically, we anticipate that the PGC system, due to springtime cover crop presence, will reduce concentrations and losses more than the interseeded cover treatments. Similarly, we expect that the growth of interseeded cover crops will significantly influence nutrient losses and concentrations in groundwater during summer and fall.

## 2 MATERIALS AND METHODS

### 2.1 Site description and water monitoring

This study was conducted at the Iowa State University Northeast Research and Demonstration Farm (42.931, −92.57), located near Nashua, Iowa, during the crop years 2021–2023. Soils at the site include Kenyon Loam (2%–5% slopes; C), Readlyn silt loam (1%–3% slopes; C/D), and Floyd loam (1%–4% slopes; B/D) (USDA NRCS, 2025). The research site consists of 36 separate plots. For this 3‐year experiment, 12 of these plots were utilized, each measuring 0.4 ha (58.5 × 67 m), with individual drainage facilitated by subsurface drainage lines at a depth of 1.2 m and spaced 28.5 m apart (Kanwar et al., 1999). The subsurface drainage line in the center of each plot flows to an individual sump equipped with a sump pump and flow meter logging all flow. A representative subsample (∼0.2% of the total flow) is collected during each pump cycle through a 5‐mm orifice and tube connected to a water sample bottle (Kanwar et al., 1999). The sample bottles are collected once weekly for analysis, and the flow meter reading is recorded at that time. Nutrient analysis of water samples was completed by Iowa State University's Water Quality Research Lab for total reactive phosphorus (TRP) and NO_3_‐N concentrations. Because no turbidity was observed, water samples were not filtered.

### 2.2 Treatments and experimental design

The 12 plots were divided into three blocks in a randomized complete block design. The control treatment was conventional corn production with no cover crop, with corn planted in standard 0.76 m rows in an east‐west orientation. The PGC treatment consisted of corn planted in standard row spacing with KBG planted between each corn row. There were two interseeded cover crop treatments with differing row spacing. The first interseeded treatment was corn planted in the standard row width, with the cover crops planted between rows in the 0.76 m space (standard row interseeded cover crop: SR‐ICC). The second interseeded treatment consisted of twin‐row corn, with corn rows planted in sets of two, 20 cm apart, with 1.3 m of spacing between sets of twin rows (wide row interseeded cover crop: WR‐ICC). Each plot was planted with corn every year (no cash crop rotation), and uniform planting density was maintained across all plots, irrespective of row spacing.

### 2.3 Management

Corn planting dates varied annually based on environmental conditions. Corn was planted on April 24, 2021, May 14, 2022, and May 2, 2023. The hybrid Pioneer P0075Q was used in 2021 and 2022, switching to Pioneer P9823Q in 2023 due to availability. The target planting population was 88,958 seeds ha^−^^1^. Approximately 224 kg ha^−^^1^ annually (200 lb ac^−^^1^) of total nitrogen fertilizer was applied to each plot through injected liquid swine manure sourced from a nearby growing‐finishing swine facility. Manure was injected approximately 15 cm below the surface in 0.76 m spacing after soil temperatures had fallen below 10°C. Plots were stripped‐tilled annually in the fall after corn harvest.

**FIGURE 1:**
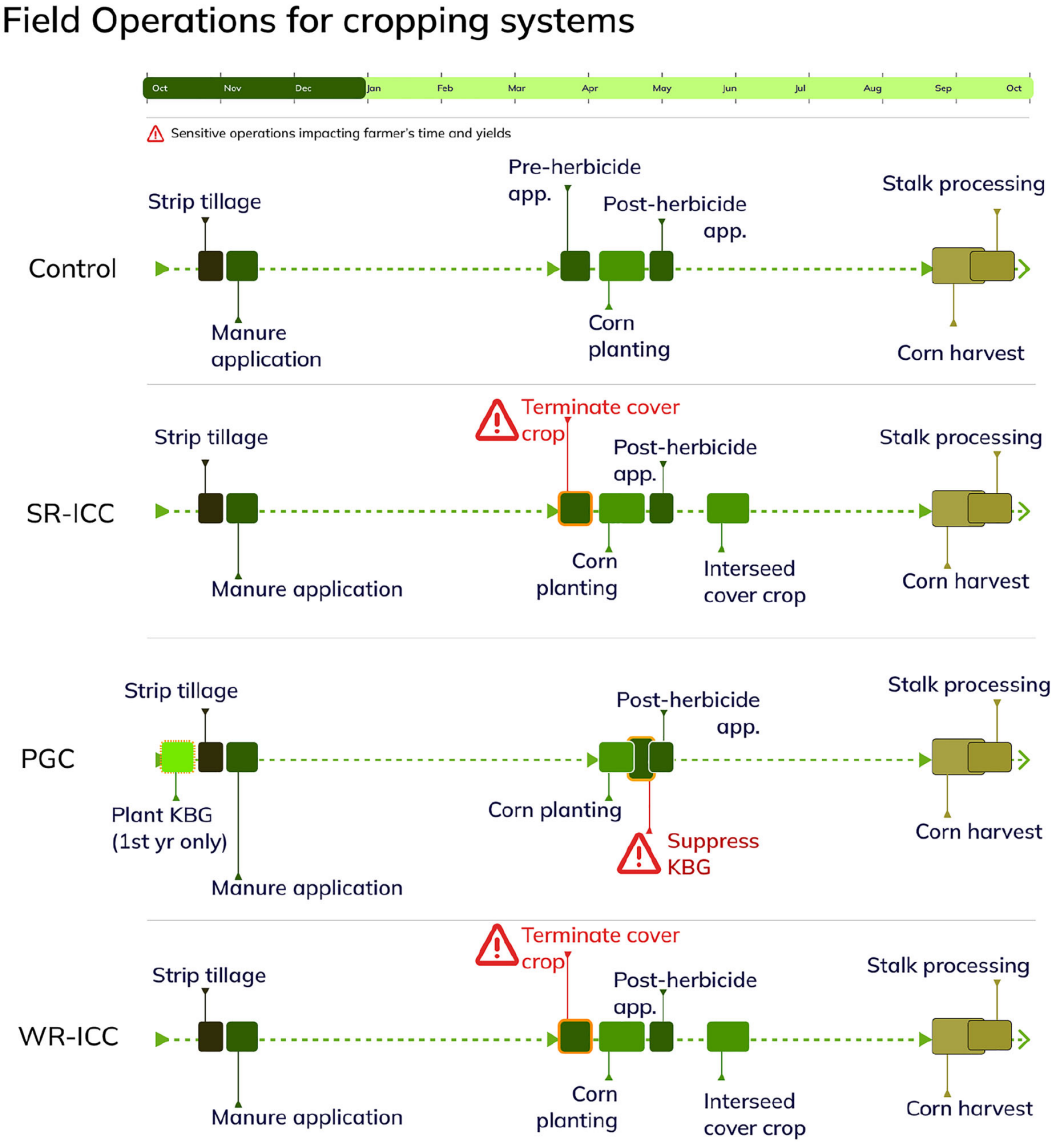
Field operations visualizer (FOV) illustrating the sequence and timing of key field operations across four experimental cropping systems: no‐cover control, standard row interseeded cover crop (SR‐ICC), perennial groundcover (PGC), and wide row interseeded cover crop (WR‐ICC).

Midnight KBG (OutsidePride, 2024) was initially planted with a grain drill on October 16, 2020, at a rate of 19 kg ha^−1^ (17 lb ac^−1^). Due to poor establishment, the KBG was broadcast planted at the same rate in June 2023. The KBG was sprayed with 96 oz ha^−1^ glufosinate in 2022 and 2023, shortly after corn planting, to limit shade avoidance responses in corn (Bartel et al., 2020). The multispecies cover crop mix was seeded at approximately V5 corn development stage in the interseeded treatments. In 2021 and 2022, the interseeded cover crops were planted with a grain drill. Due to a lack of grain drill availability, the interseeded cover crops were broadcast planted in 2023. The cover crop mix varied based on the availability of seed, as shown in Table 1. The dates of interseeding were June 8, 2021, June 17, 2022, and June 8, 2023. The interseeded cover crop species were not winter hardy. Timing and sequence of operations are illustrated in Figure 1 using the Field Operations Visualizer (Rockson et al., 2024).

**TABLE 1:** Interseeded cover crop mix.

Species	2021	2022	2023
	Seeding rate (kg ha^−1^)		
*Crotalaria juncea* L.	3.5	3.5	3.4
*Vigna unguiculata* (L.) Walp. ‘CatJang black’	5.7	5.7	–
*Melilotus officinalis* (L.) Lam.	2	–	1.2
*Fagopyrum esculentum Moench* ‘Mancan’	5.7	5.7	5.0
*Lolium multiflorum* Lam. ‘Tam Tbo’	3.5	3.5	3.7
*Phacelia tanacetifolia* Benth. ‘Super Bee’	2.3	2.3	2.2
*Brassica juncea* (L.) Czern. ‘Kodiac’	1.3	1.3	–
*Brassica napus* L. ‘Trophy’	1.1	1.1	1.1
*Vicia villosa* Roth ‘MT’	3.8	3.8	3.4
*Brassica juncea* (L.) Czern. ‘Indi Gold’	–	–	1.3
*Vigna unguiculata* (L.) Walp. ‘Iron & Clay’	–	–	9.2
Total	29	28	30

### 2.4 Data collection and statistical analysis

Corn grain yield was calculated as the average of three measurement locations within each plot, adjusted to 15% moisture content. Total drainage depth was calculated by dividing the total flow volume by the drainage area (50% of the plot area, or 1960 m^2^ per plot). Annual flow‐weighted average (FWA) nutrient concentrations were calculated by summing the total nutrient mass loss for each plot and dividing by total subsurface drainage flow. In each year, there were periods of time where there was flow in a weekly period, but the volume was too low to collect a sample for nutrient analysis. The overall proportion of annual flow was low for these periods (<1%). Above‐ground biomass samples were collected from approximately 4 m^2^ sections of the interseeded cover crop plots each year shortly following corn harvest. These samples were dried and weighed to determine yield without separating cover crop species.

A frequency grid consisting of 25 squares (5 × 5), each 15 cm × 15 cm, was used in the spring of each year to quantify the ground coverage of the PGC and help illustrate the success of establishment at the start of the season (Vogel & Masters, 2001). The grid was placed in four random places within the grass strip in each plot, and then the number of squares that had green grass evident was counted to estimate the percentage presence of cover.

For seasonal analysis, spring was defined as March–June, summer as July–September and fall as October–December. There was no tile flow before March in any year. Annual grain yield, annual FWA NO_3_‐N and TRP concentrations, annual NO_3_‐N and TRP losses, and annual drainage flow data were analyzed using a repeated measures approach in SAS (PROC MIXED). For annual models, treatment, year, and the treatment × year interaction were treated as fixed effects. For seasonal analyses of NO_3_‐N, season and the treatment × season interaction were additionally included as fixed effects. Block and the block × year interaction were treated as random effects. Year was specified as the repeated measure with the subject defined as the block × treatment interaction, utilizing appropriate covariance structures (e.g., compound symmetry) and the Kenward‐Roger approximation to calculate denominator degrees of freedom. To evaluate significant interactions, a SLICE statement was utilized to test for treatment effects within individual years or seasons. Pairwise comparisons of the least squares means were generally performed using Fisher's least significant difference, with Dunnett's adjustment applied specifically to the seasonal nitrate models to compare cover crop treatments against the no‐cover control. Due to the high coefficients of variation, statistical tests were evaluated at the *α* = 0.1 significance level. A complete summary of the *p*‐values for all main effects and interactions evaluated in these models is provided in Table S1. Cover crop establishment metrics, including spring PGC ground coverage and fall above‐ground biomass, were evaluated using descriptive statistics (means, ranges, and standard errors).

## 3 RESULTS AND DISCUSSION

### 3.1 Precipitation and subsurface drainage flow

Growing season precipitation measured by a rain gauge at the farm was below the long‐term average in each year (Table S2). In the critical May–July period, precipitation was 49%, 19%, and 68% lower than average for 2021, 2022, and 2023, respectively. Precipitation was lowest in 2023, with the total growing season precipitation 42 cm lower than the long‐term average.

No evidence of treatment effects (Table 2) was observed in 2021 or 2022 for total annual drainage depth. In 2023, the PGC treatment had a greater average annual drainage flow than the other three treatments. On average, 2022 saw the highest drainage flows, while 2023 saw the lowest. Differences among treatments in annual drainage flow were less evident (*p* = 0.58) analyzing the results over the 3‐year period. The annual drainage flows in these plots ranged from 47% to 55% of the lowest observed for these plots from 2008 to 2015 by Dougherty et al. (2020). The average proportion of growing season precipitation that became drainage in this study was 5.4%, 18%, and 0.7% in 2021–2023, respectively.

**TABLE 2:** Average annual subsurface drainage depth (cm) by treatment and year.

Treatment	2021 (cm)	2022 (cm)	2023 (cm)	3‐Year average (cm)
No‐cover control	3.09a	12.6a	0.036a	5.24a
PGC	4.73a	12.4a	0.57b	5.89a
SR‐ICC	3.54a	12.1a	0.19a	4.97a
WR‐ICC	3.44a	11.2a	0.21a	5.28a
Average	3.70A	12.1B	0.25C	

*Note*: Lowercase letters indicate statistical differences among treatments within a given year. Uppercase letters indicate statistical differences among the annual averages across all 3 years (*p* ≤ 0.1)

Abbreviations: PGC, perennial groundcover; SR‐ICC, standard row interseeded cover crop; WR‐ICC, wide row interseeded cover crop.

### 3.2 Groundcover establishment and annual cover crop biomass

Quantifying the establishment of the PGC proved difficult due to the variability in each plot, within and between rows. Images are provided in Table S3 to illustrate the establishment of each cover crop treatment. As summarized in Table 3, overall cover crop establishment varied drastically across treatments and years. Generally, the PGC establishment was poor and patchy. The drier‐than‐normal conditions after the Fall 2020 planting resulted in very limited grass growth. The PGC became more robust in early 2022, but it did not recover to the same level after suppression. When the KBG was replanted in June of 2023, precipitation was below average for the rest of the growing season, and grass establishment was still poor. The average PGC coverage in the grass strip in the spring ranged from 3.5% to 14.3%, which translates to grass covering 1.8%–7% of the total area of the plots.

**TABLE 3:** Annual cover crop establishment and biomass production by treatment.

Year	PGC spring coverage (%)	Interseeded cover crop biomass, kg DM ha^−1^	
		Standard row	Wide row
2021	3.5 (0–9)	14.7 (5.33)	1090 (142)
2022	14.3 (4–24)	21.2 (6.97)	1410 (471)
2023	7.2 (0–18)	47.0 (29.7)	1090 (78.3)

*Note*: Average Kentucky bluegrass perennial groundcover (PGC) coverage measured in the grass strip during the spring (range in parentheses), and average above‐ground biomass yield for the standard row (SR‐ICC) and wide row (WR‐ICC) interseeded cover crop treatments measured in the fall (standard error of the means in parentheses). Spring PGC measurement dates were April 30, 2021, May 20, 2022, and April 29, 2023.

For the interseeded annual systems, Table 3 demonstrates that cover crop establishment and growth were most successful in the WR‐ICC treatment each year, which produced over 50 times more above‐ground biomass than the SR‐ICC treatment. The large difference in biomass production between row widths is consistent with what has been found in other studies (Black et al., 2023; Johnson, 2020). The WR‐ICC treatment produced more biomass on average than cereal rye (*Secale cereale* L.), the most popular annual winter cover crop in Iowa (Singer, 2008), in a previous study at the same site, while the SR‐ICC cover produced less (Dougherty et al., 2020). Biomass production is critical to NO_3_‐N loss reduction (Christianson et al., 2021), so cover crops that produce more biomass without impacting crop yield would be desired.

### 3.3 Seasonal nitrate concentrations

Analysis of NO_3_‐N concentrations showed evidence of a three‐way interaction between treatment, season, and year (*p* < 0.001), indicating that the effectiveness of the cover crop systems depended on the season, and this seasonal pattern was itself altered by the year's specific weather conditions. The timing of the highest nitrate concentration and mass loss varied between years, showing the influence of precipitation on nutrient transport in tile‐drained plots.

The low drainage in 2021 (3.7 cm) limited nitrate transport, though some treatment differences appeared. In the spring of 2021 (Table 4), the SR‐ICC treatment had higher nitrate concentrations (10.8 mg L^−^^1^) than the control. This likely reflected residual nitrate remaining at depth from the prior spring‐applied manure rather than new mineralizable N. Because these plots had less time for post‐application drainage than the fall‐applied treatments, a portion of the nitrate likely persisted in the profile and was gradually expressed in early 2021 tile flow as the soil rewetted. Later‐season rainfall favored the WR‐ICC treatment, whose greater biomass reduced summer concentrations by over 80% and fall concentrations by nearly 60% relative to the control.

**TABLE 4:** Seasonal and annual NO_3_–N concentration in the subsurface water.

Year	Season	No‐cover (mg N L^−1^)	PGC (mg N L^−1^)	SR‐ICC (mg N L^−1^)	WR‐ICC (mg N L^−1^)
2021	Spring	7.22b	7.60b	10.81a	6.39b
	Summer	16.14a	4.83b	2.46c	2.61c
	Fall	5.45a	4.33a	3.72ab	2.21b
	Annual	9.60A	5.59A	5.66A	3.74A
2022	Spring	16.71a	12.49b	15.23a	14.07a
	Summer	26.36a	18.92c	23.79ab	22.10b
	Fall	–	–	–	–
	Annual	21.54B	15.71B	19.51B	18.09B
2023	Spring	9.70a	7.09a	8.89a	6.87a
	Summer	–	–	–	–
	Fall	–	–	–	–
	Annual	9.70A	7.09A	8.89A	6.87A
Average	Spring	11.06	8.26	9.20	8.26
	Summer	20.58	11.73	14.14	11.89
	Fall	9.64	8.25	10.17	7.67
	Overall average	13.76	9.41	11.17	9.28

*Note*: The 3‐year average shows differences between the no‐cover control, standard row interseeded cover crop (SR‐ICC), wide row interseeded cover crop (WR‐ICC), and Kentucky bluegrass perennial groundcover (PGC). Lowercase letters indicate significant differences among treatments within a given year or season (*p* ≤ 0.1). Uppercase letters indicate significant differences among the annual averages across all 3 years (*p* ≤ 0.1).

In 2022, sufficient drainage (12.1 cm) reconnected the manure application zone with the tile drains, making treatment effects evident. Nitrate concentrations peaked in summer, with the no‐cover control reaching 26.4 mg L^−^^1^, while the PGC averaged 18.9 mg L^−^^1^ (*p* < 0.001). The PGC system was also highly effective in spring, where it had the numerically lowest nitrate concentration (12.5 mg L^−^^1^) and was significantly lower than the no‐cover control. Despite producing much more biomass, the WR‐ICC did not improve water quality relative to the SR‐ICC, likely because the higher‐yielding SR‐ICC corn (2.2 Mg ha^−^^1^ greater) compensated through greater nitrogen uptake. In the 2023 drought year, minimal drainage (0.25 cm) prevented nitrate movement to the tiles, explaining the lack of measurable treatment differences (*p* = 0.45).

**FIGURE 2:**
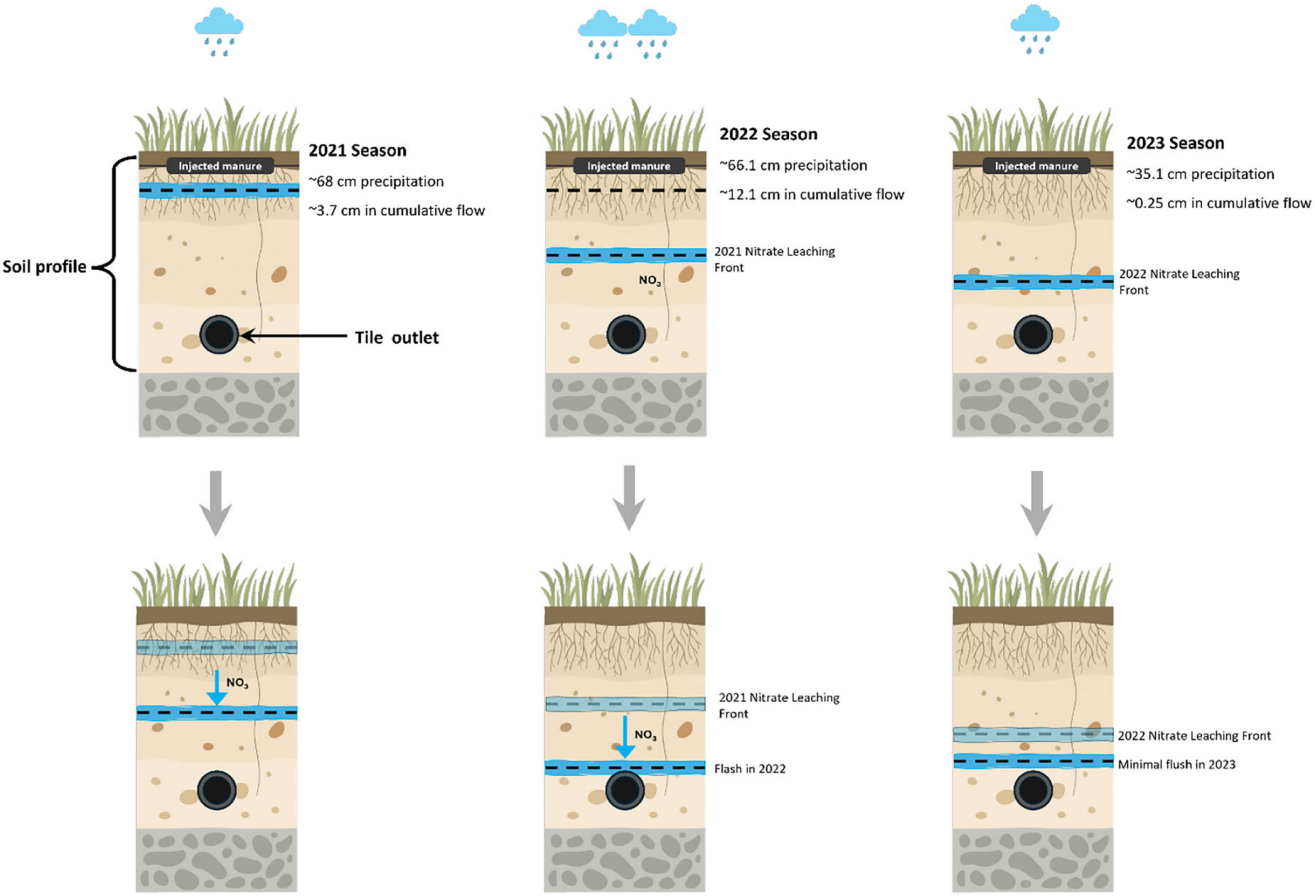
A conceptual diagram illustrating how annual precipitation and cumulative subsurface drainage (flow) influenced vertical nitrate (NO_3_^−^) transport during the 2021, 2022, and 2023 seasons.

The observed treatment effects can be attributed to differences in vertical nitrate transport, which are visually conceptualized in Figure 2. Because manure was injected at a shallow depth (∼15 cm) and tile drains were installed significantly deeper (∼120 cm), a substantial volume of percolating water was required to move the nitrogen through the soil profile. As illustrated in the figure, the “wet” 2022 season provided enough cumulative drainage (12.1 cm) to mobilize residual nitrate into the tiles, whereas the drought conditions of 2023 resulted in minimal vertical movement and a lack of treatment differences. This relationship between drainage volume and nitrate export is also documented in the study Kladivko et al. (2004).

### 3.4 Flow‐weighted concentrations and annual nitrate losses

Across the 3‐year period, flow‐weighted nitrate concentrations showed a consistent numerical reduction in all cover crop treatments relative to the control. The PGC (9.07 mg L^−^^1^) and WR‐ICC (8.87 mg L^−^^1^) averaged 20% and 22% lower than the no‐cover control (11.32 mg L^−^^1^), respectively. The treatment effect (*p* = 0.27) and the treatment × year interaction (*p* = 0.25) provide no statistical evidence for consistent differences among treatments across years, suggesting that these reductions were modest and variable over time. In 2021, stronger evidence of a treatment effect was observed (*p* < 0.01), with the WR‐ICC exhibiting the lowest NO_3_–N concentration.

Nitrogen losses were highest in 2022 across all treatments, coinciding with peak drainage volumes and a spring‐dominant precipitation pattern that increased leaching risk during early crop development (Jaynes et al., 1999; Waring et al., 2020). The PGC treatment had the greatest NO_3_‐N losses (Table S4) and annual drainage flow in 2021 (2.36 ± 0.35 kg N ha^−^^1^) and 2023 (0.42 ± 0.22 kg N ha^−^^1^). The PGC system's performance on N losses varied by year, reflecting its establishment challenges. In its first year, 2021, the PGC treatment had the highest N loss (2.36 kg N ha^−^^1^), larger than the other cover crop systems, which likely reflects limited root development and N uptake (Thilakarathna et al., 2016; Tonitto et al., 2006). On the contrary, in 2022, the year with the best PGC ground coverage, the PGC system had the lowest numerical N loss (18.0 kg N ha^−^^1^), resulting in a 2.1%–20.3% reduction compared to the other treatments over the 3 years. However, due to the high overall variability in that high‐drainage year, there was little statistical evidence for this difference in total annual loss. The 20% nitrate reduction observed in our field study aligns closely with the 14% reduction predicted by Bartel et al. (2020) using APSIM simulations, supporting that the water‐quality benefits of KBG PGC extend from modeled to field conditions. Although not statistically evident, the 3‐year average NO_3_‐N loss was 20% lower in PGC than in the control, consistent with their modeling.

Taken together, these results explain key aspects of nutrient transport in a variable climate. The high summer nitrate concentrations in 2022, following a drier year, illustrates how residual soil nitrate can accumulate and then flush out in a single, large pulse during high‐drainage events. While overall nitrate losses during our study were lower than those reported in wetter periods, the timing of this flush is a critical distinction from historical patterns where most drainage occurs in spring. As precipitation patterns shift, such large summer leaching events could become increasingly significant contributors to the Gulf hypoxic zone. A cover cropping system, which remains year‐round and can scavenge excess nitrogen throughout the summer, appears to be a promising strategy to mitigate these critical nitrate losses. However, our PGC performance remained variable throughout the study, with flow‐weighted nitrate concentration reductions ranging from negligible in the dry 2023 season to over 20% in the summer of 2022. Given the establishment challenges observed and the numerical increase in N loss during the initial year (2021), these results suggest that while the system shows potential for late‐season mitigation, further multi‐year research is needed to confirm its reliability and long‐term water quality benefits under diverse climatic conditions.

### 3.5 TRP concentrations and annual losses

Concentrations of TRP in subsurface drainage were highly variable, with little evidence of a consistent main effect for treatment (*p* = 0.84), season (*p* = 0.62), or their interactions. Instead, the concentrations were primarily driven by yearly conditions (*p* = 0.06), largely due to the 2023 crop season, which produced distinct annual FWA concentrations.

Annual FWA TRP concentrations (Table 5) varied over the 3‐year period, with a significant year effect linked to nutrient accumulation and later transport (*p* < 0.01). The lowest concentration occurred in 2022 (7.2 µg P L^−^^1^) and the highest in 2023 (244.9 µg P L^−^^1^, *p* < 0.01). Across all years, mean concentrations did not differ significantly between treatments (*p* = 0.18), consistent with Dougherty et al. (2020), who found no treatment effect in an 8‐year study of tile nutrient flows from multiple Midwestern cropping systems. PGC had lower TRP concentrations than the control (*p* = 0.27) and WR‐ICC (*p* = 0.06), with a numerical reduction compared to SR‐ICC (53% reduction, *p* = 0.44). Across all years, PGC in continuous corn reduced the 3‐year average TRP concentration by approximately 70% relative to the control.

**TABLE 5:** Annual flow‐weighted average total reactive phosphorus (TRP) concentrations from water samples analyzed.

Treatment	2021 (µg P L^−1^)	2022 (µg P L^−1^)	2023 (µg P L^−1^)	3‐Year average (µg P L^−1^)
No‐cover control	12.3	6.3	299.3b	81.8
PGC	9.6	7.1	57.6a	24.8
SR‐ICC	7.0	5.6	145.2ab	52.6
WR‐ICC	11.1	9.8	495.8 c	172.2
Annual average	10.0A	7.2A	244.9B	

*Note*: Lowercase letters indicate significant differences among treatments within a given year (*p* ≤ 0.1). Uppercase letters indicate significant differences between years within a specific treatment (*p* ≤ 0.1). For the Annual Average row, uppercase letters denote significant differences across the study years. The 3‐year average shows differences between the no‐cover control, standard row interseeded cover crop (SR‐ICC), wide row interseeded cover crop (WR‐ICC), and Kentucky bluegrass perennial groundcover (PGC) in TRP concentrations.

Although TRP concentrations were highest in 2023, total annual TRP losses (Table S5) were lowest due to minimal drainage flow. The highest annual loss occurred in 2022, which also had the greatest drainage. This contrast between high concentrations and low losses reflects TRP's limited mobility compared to nitrate (NO_3_^−^), as it binds strongly to soil particles and requires substantial percolation to reach tile drains (Tesoriero et al., 2009). Low precipitation in preceding months likely allowed TRP to accumulate in the soil; when drainage occurred in 2023, this produced a high concentration “flush,” but total P loss remained small.

Historical management may also have influenced treatment responses. The WR‐ICC plots, which recorded the highest TRP concentrations in 2023, previously received gypsum applications (Dougherty et al., 2021), which may have altered P dynamics (Osterholz et al., 2020, 2023). These legacy effects, combined with hydrologic variability, show the need for multi‐year monitoring to capture treatment impacts on TRP transport.

### 3.6 Grain yield

The average yields (Table 6) for the study period indicate that both year and treatment had an influence on grain yield (*p* < 0.001) as well as their interaction (*p* = 0.06). The effect of year was evident in the yield variability, with the highest average yield occurring in 2021 (13.2 Mg ha^−^^1^) and the lowest in 2023 (9.52 Mg ha^−^^1^). The yield, particularly in 2023, which was below county and state averages, can be attributed to severe drought conditions, as multiple studies have documented the negative impact of water deficits on kernel development and crop yields (Claassen & Shaw, 1970; Gaudin et al., 2015; Li et al., 2018).

**TABLE 6:** Average corn grain yields for the years 2021–2023, adjusted to 15% moisture content.

Treatment	2021 (Mg ha^−1^)	2022 (Mg ha^−1^)	2023 (Mg ha^−1^)	3‐Year average (Mg ha^−1^)
No‐cover control	14.3a	12.8a	10.1a	12.4a
PGC	13.6a	13.1a	10.1a	12.3a
SR‐ICC	14.0a	12.9a	10.0a	12.3a
WR‐ICC	10.9b	11.4b	7.93b	10.1b
Annual average	13.2a	12.6b	9.52c	

*Note*: Letters indicate results of pairwise comparisons within each year; the means with the same letter showed no strong evidence of difference (*p* ≤ 0.1).

Abbreviations: PGC, perennial groundcover; SR‐ICC, standard row interseeded cover crop; WR‐ICC, wide row interseeded cover crop.

The WR‐ICC treatment had the lowest 3‐year average yield at 10.1 Mg ha^−1^ which was the primary reason for differences between treatments—Control (12.4 Mg ha^−^^1^), PGC (12.3 Mg ha^−^^1^), and SR‐ICC (12.3 Mg ha^−^^1^). Notably, the averages from the other three treatments are comparable to the Floyd County, Iowa's, and the state's average of 12.6 Mg ha^−1^ for the 2021–2023 timeframe (Johanns, 2024).

Yield responses to the WR‐ICC treatment varied notably among years (treatment × year, *p* = 0.04), indicating that yield effects depended on seasonal conditions. During the dry springs of 2021 and 2023, WR‐ICC yields were approximately 24% lower than the other treatments, whereas in the wetter spring of 2022, the yield gap narrowed to about 13%. This is likely reflecting differences in early‐season soil moisture availability and crop competition with the interseeded cover across years. In the years with dry spring seasons, corn in the WR‐ICC plots failed to fully canopy, potentially worsening drought stress due to increased evaporation from the exposed soil surface. Additionally, under drought conditions, corn roots tend to grow less laterally as they search for moisture deeper in the soil, which might pose a challenge in the WR‐ICC treatment where manure was applied in standard row widths, requiring lateral root development to access nitrogen fully (Kang et al., 2022). Furthermore, competition for moisture and nutrients from vigorous cover crops may have contributed to the observed yield reduction.

The average yield loss of the WR‐ICC treatment was 2.3 Mg ha^−1^, compared to the control, equivalent to an annual loss of $540 ha^−1^ based on 2023 average Iowa corn prices (Johanns, 2025). Despite this direct economic trade‐off, the substantial above‐ground biomass production in the WR‐ICC system—which exceeded 1000 kg DM ha^−^^1^ in two out of 3 years—may offer long‐term agronomic and environmental advantages, such as enhanced soil organic matter and forage production, that are not captured by grain yield alone. While yield results are promising for the PGC treatment, grass establishment was poor, and more robust grass may result in lower grain yields, especially if the grass is not properly suppressed (Galland et al., 2022).

### 3.7 Relationships between precipitation, tile flow, biomass, coverage, nutrient reduction, and yield

The outcomes of this 3‐year study were impacted by the below‐average precipitation, which limited both drainage and cover crop establishment. This variation in water flux was the primary factor controlling annual nutrient loads and grain yields. The performance of each cover crop system highlighted a different response to these water‐limited conditions. The PGC system establishment was poor (14.3% coverage in its best year), which likely influenced the variability of the observed results. Despite this limited stand, the system showed a reduction in NO_3_‐N and TRP concentrations in specific seasons, such as the summer of 2022, but these reductions were not uniform across the 3‐year study period. In contrast, the WR‐ICC system consistently produced substantial biomass, resulting in the best water quality benefits, but at the cost of a consistent 20% average yield reduction compared to the control. The SR‐ICC system achieved numeric water quality benefits, sometimes lower but without the yield penalty observed in the wide‐row configuration; therefore, this treatment may warrant more focused attention in future research as a lower risk alternative for farmers.

These results show a critical trade‐off under the study's dry conditions: the high‐biomass annual system improved water quality at a direct cost to yield, while the low‐biomass perennial system offered the potential for water quality benefits without harming yield, though its primary challenge remains successful establishment in a variable climate. The performance of each system was highly context‐dependent, reflecting a direct trade‐off between biomass production, nutrient scavenging, and cash crop competition under water‐limited conditions.

## 4 CONCLUSIONS

This study highlighted the challenge of implementing novel cover crop systems under unfavorable, dry precipitation conditions. Despite limited grass establishment, the PGC system reduced NO_3_‐N FWA concentrations by 20% (with a corresponding 27% reduction in annual nitrogen loads) relative to the no‐cover crop control, with no significant difference in grain yield detected. The WR‐ICC was more effective in reducing NO_3_‐N concentrations (22% lower than the control) due to greater cover crop growth, but this benefit was offset by a substantial reduction in corn yield. While the WR‐ICC treatment currently faces a high yield trade‐off, its high biomass production was notable; with optimized nutrient placement and species selection, it may offer the most effective long‐term strategy for nutrient reduction, as greater evapotranspiration from active biomass can further reduce drainage and associated leaching. The lack of yield penalty observed in the SR‐ICC treatment suggests it remains a viable, lower‐risk configuration that warrants further investigation, particularly for farmers prioritizing agronomic consistency. Also, treatment effects on seasonal NO_3_‐N concentrations were observed, but these effects were highly dependent on the specific weather conditions of each year and season. The treatment differences were not statistically detectable for annual drainage, N loads, or TRP loads except in 2023, the driest year. Crucially, this study revealed that the period of highest nitrate leaching risk can shift from spring to summer, particularly following a dry year, which has significant implications for nutrient management. Because weather conditions remain beyond farmer control, this study provides valuable insight into the impacts of drought on these systems. Further research is needed to refine PGC planting and management practices and to develop robust methods for quantifying PGC establishment at larger scales.

## AUTHOR CONTRIBUTIONS

**Gabrielle M. Myers‐Bailey**: Data curation; formal analysis; investigation; methodology; resources; validation; writing—original draft; writing—review and editing. **Philip E. Rockson**: Data curation; formal analysis; investigation; validation; visualization; writing—review and editing. **Daniel S. Andersen**: Conceptualization; funding acquisition; methodology; resources; supervision; validation; writing—review and editing. **Kenneth J. Moore**: Methodology; validation; writing—review and editing. **Matthew J. Helmers**: Validation; writing—review and editing. **D. Raj Raman**: Conceptualization; funding acquisition; resources; supervision; writing—review and editing.

## CONFLICT OF INTEREST STATEMENT

The authors declare no conflicts of interest.

## Supporting information



Supplementary Material

## Data Availability

The dataset for this experiment can be found under the perennial groundcover consortium in Zenodo. These data are publicly available at https://doi.org/10.5281/zenodo.18972358.
