# Supplementary material for: Assessing the impact of perennial groundcover and interseeded annual cover crops on maize yield and drainage water quality
Source: J Environ Qual. 2026 Apr 2;55(2):e70175. doi: 10.1002/jeq2.70175 (PMC13047304; doi:10.1002/jeq2.70175)
Supplement: Supplementary file 1 — Supplementary Material [file JEQ2-55-0-s001.docx]

supplemental material

Supplemental Table S1. Analysis of variance (ANOVA) summary of P-values for fixed effects on annual nutrient concentrations, nutrient loads, corn grain yield, and seasonal nitrate loss.

| **Dependent Variable** | **Treatment** | **Year** | **Season** | **Treatment × Year** | **Treatment × Season** |
| --- | --- | --- | --- | --- | --- |
| Grain Yield (Annual) | < 0.01 | < 0.01 | — | 0.06 | — |
| FWA NO₃-N (Annual) | 0.27 | < 0.01 | — | 0.25 | — |
| NO₃-N Load (Annual) | 0.35 | < 0.01 | — | 0.03 | — |
| FWA TRP (Annual) | 0.182 | < 0.01 | — | 0.20 | — |
| TRP Load (Annual) | 0.41 | < 0.01 | — | 0.64 | — |
| Nitrate (Seasonal) | 0.04 | < 0.01 | < 0.01 | 0.21 | < 0.01 |

Note: Bold values indicate statistical significance at the **α** = 0.1 level. Dashes (—) indicate that the effect was not included in the respective model

Supplemental Table S2. Growing season precipitation (cm) for 2021-2023 compared to the 1994-2023 average.

|  | **2021** | **2022** | **2023** | **1994-2023 Average** |
| --- | --- | --- | --- | --- |
| April | 1.60 | 9.19 | 4.70 | 9.12 |
| May | 8.84 | 10.4 | 4.90 | 11.9 |
| June | 3.61 | 13.3 | 4.14 | 14.7 |
| July | 6.43 | 6.48 | 2.97 | 10.6 |
| August | 26.9 | 17.1 | 3.81 | 11.3 |
| September | 4.09 | 2.62 | 8.38 | 8.82 |
| October | 11.43 | 1.91 | 5.49 | 6.97 |
| November | 5.13 | 5.13 | 0.74 | 3.88 |
| **Total** | **68.0** | **66.1** | **35.1** | **77.3** |
| *Total Deviation from Average* | -9.26 | -11.1 | -42.1 |  |

**
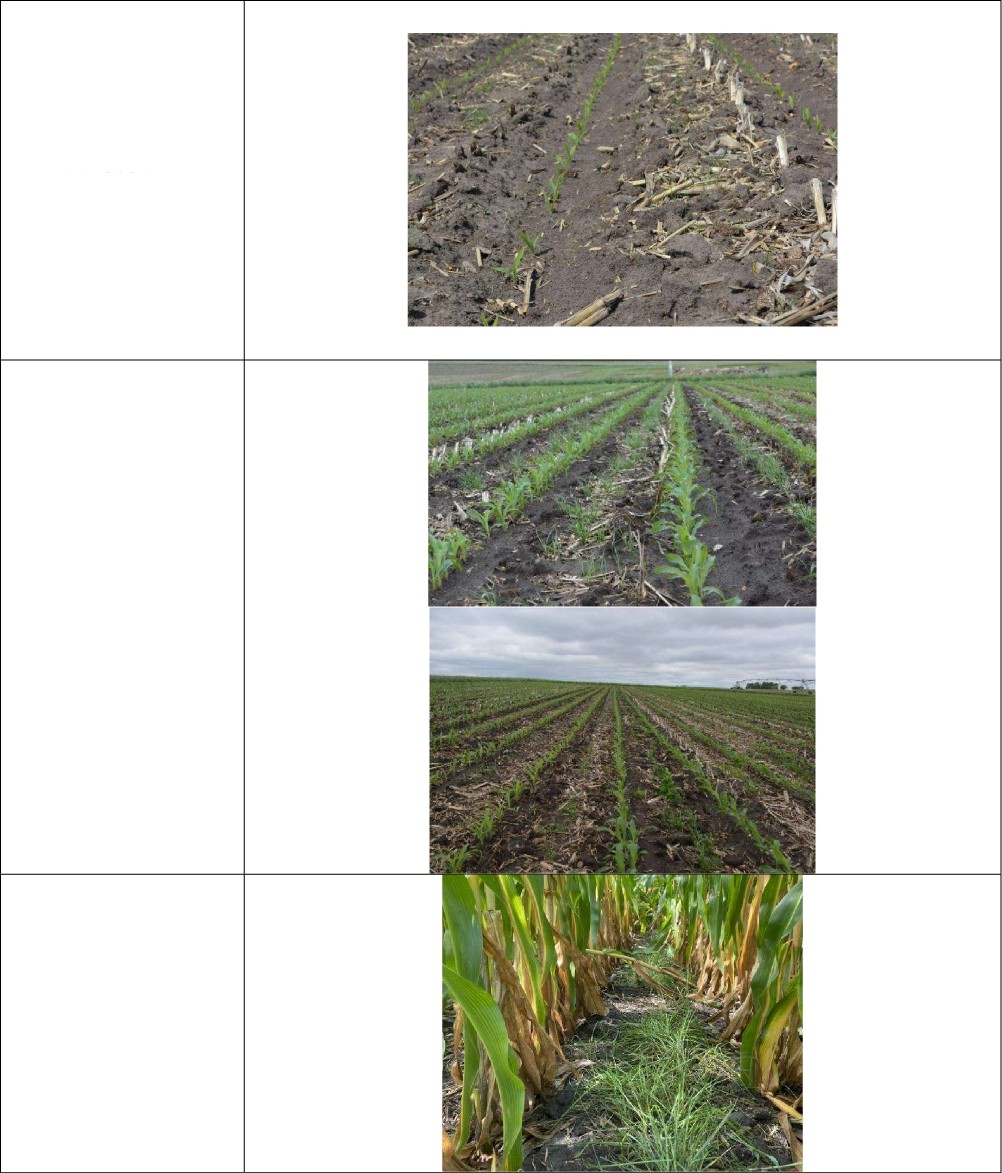
Supplemental Table S3. Photos from cover cropped treatments.**

Photos from perennial groundcover plots

May 14, 2021

May 28, 2021

August 27, 2021


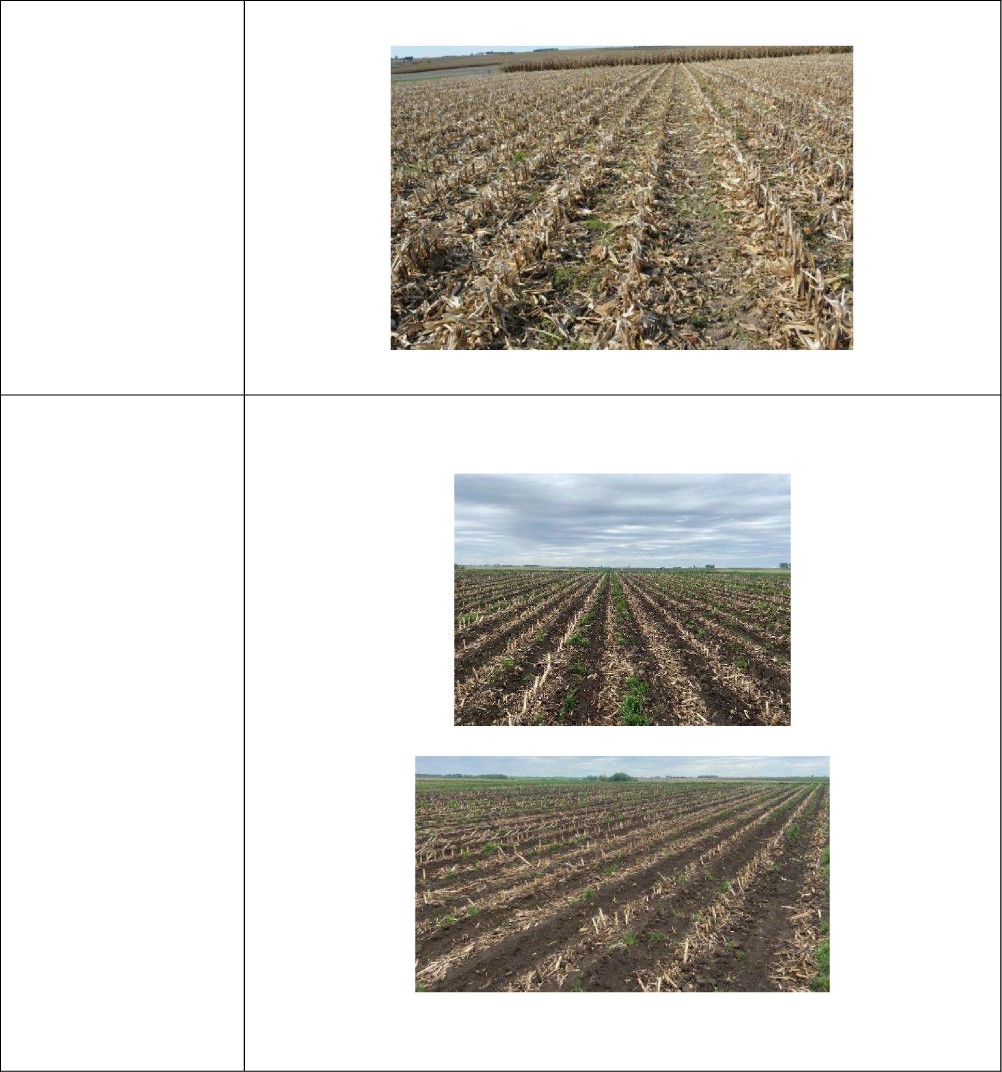


October 15, 2021

May 20, 2022


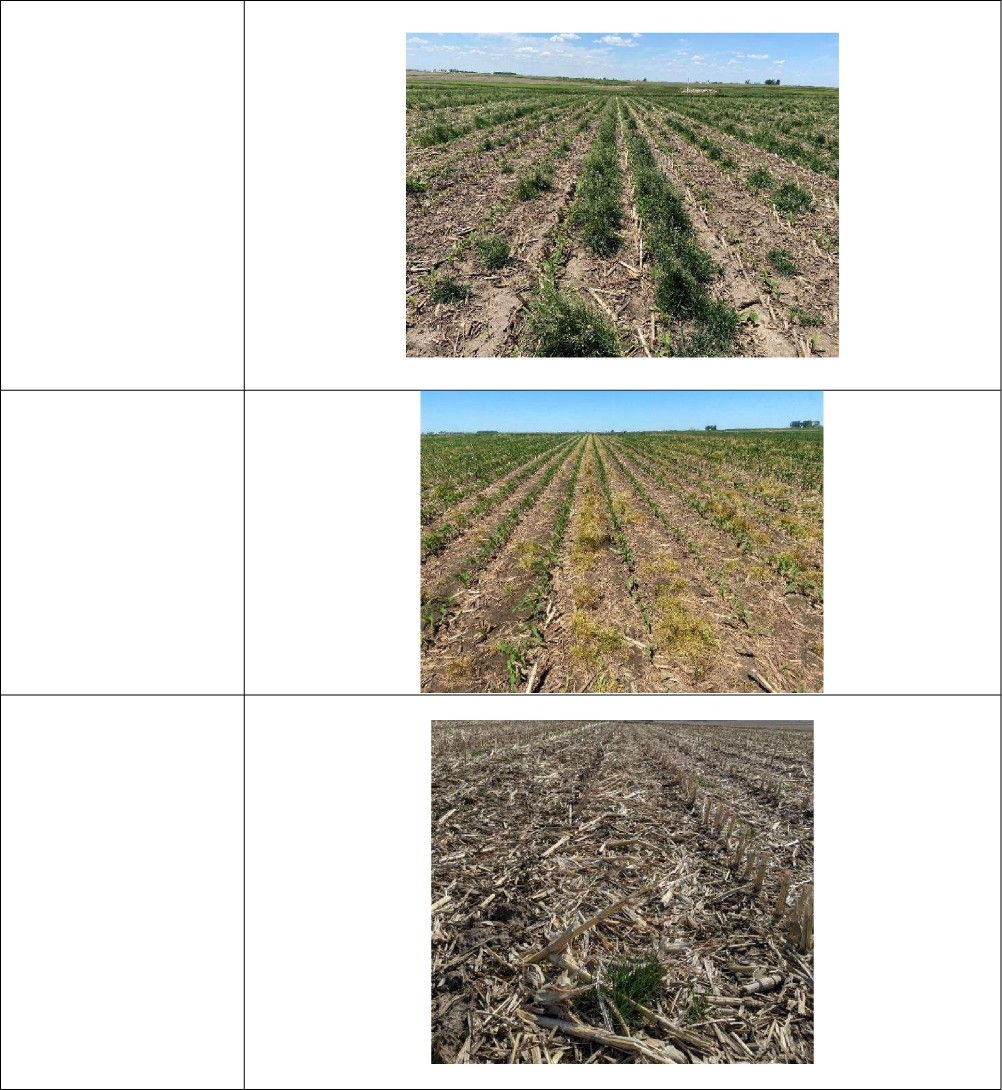


June 9, 2022

June 16, 2022 (post- suppression)

April 28, 2023


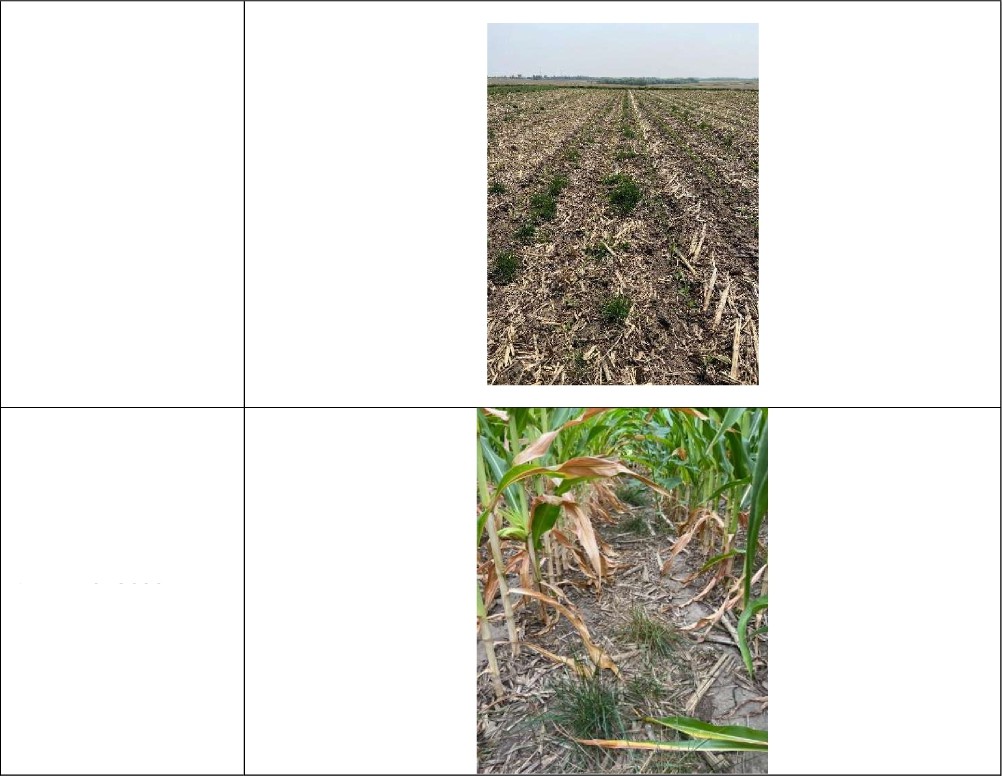


May 23, 2023

August 3, 2023


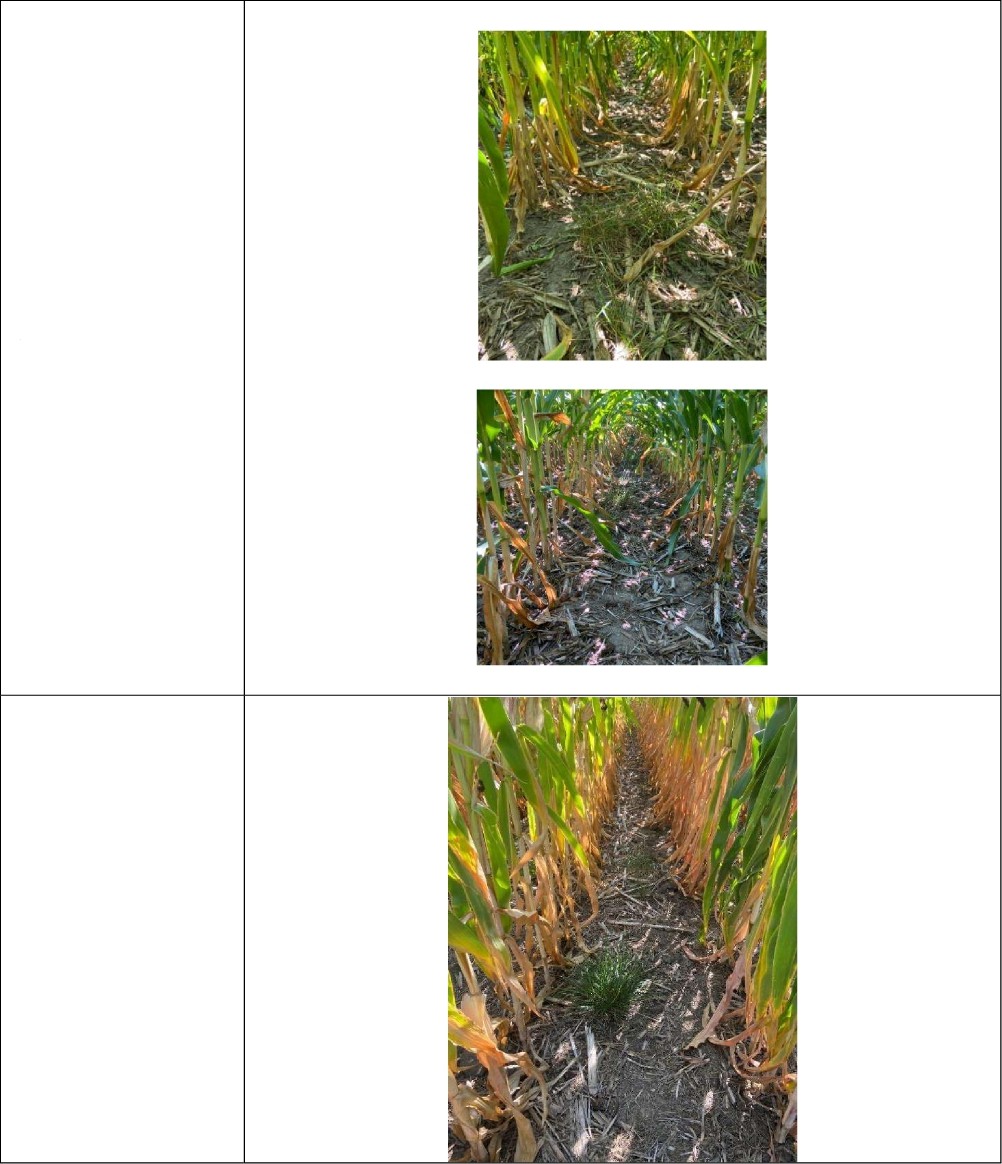


August 17, 2023

September 1, 2023


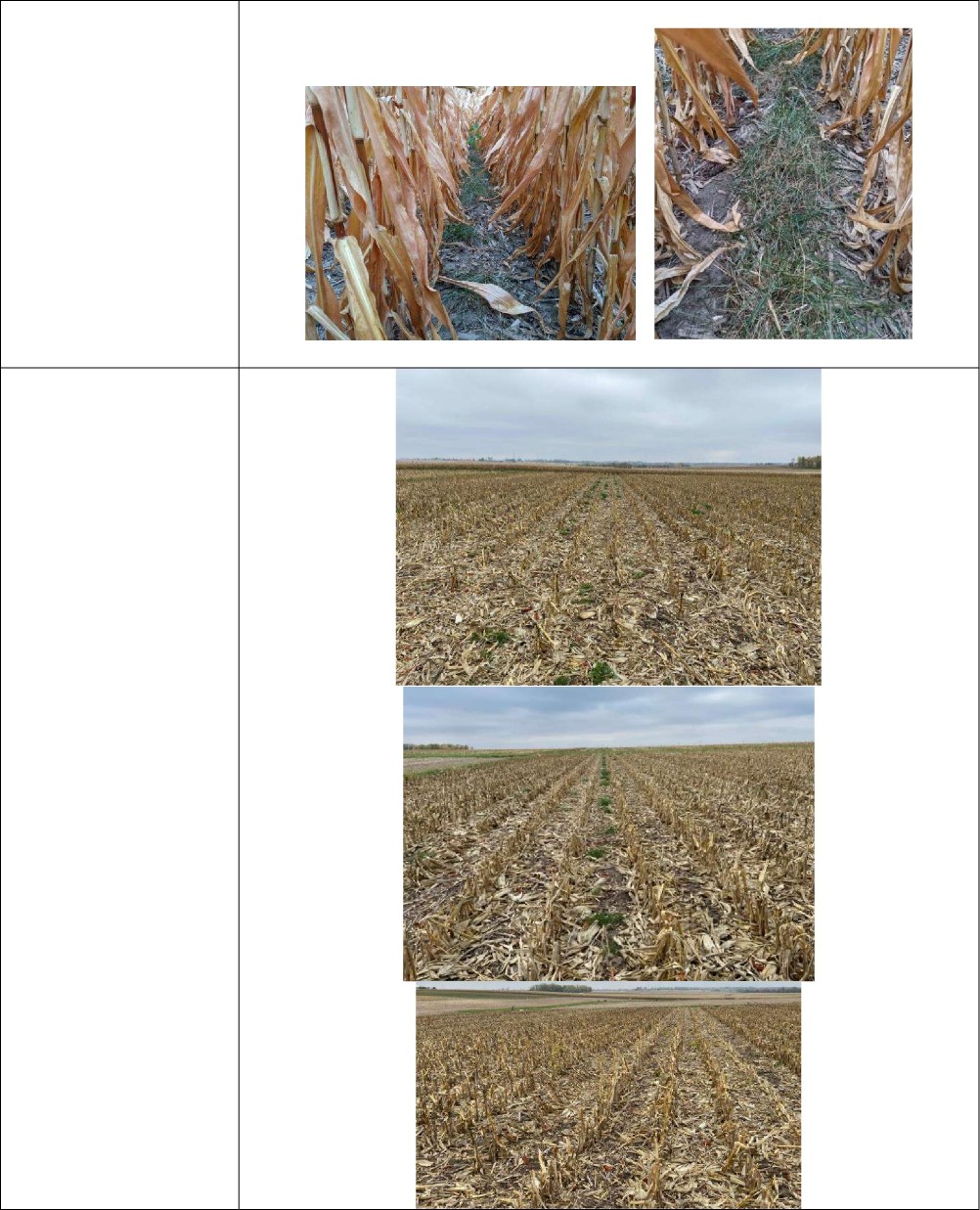


September 15, 2023

October 20, 2023


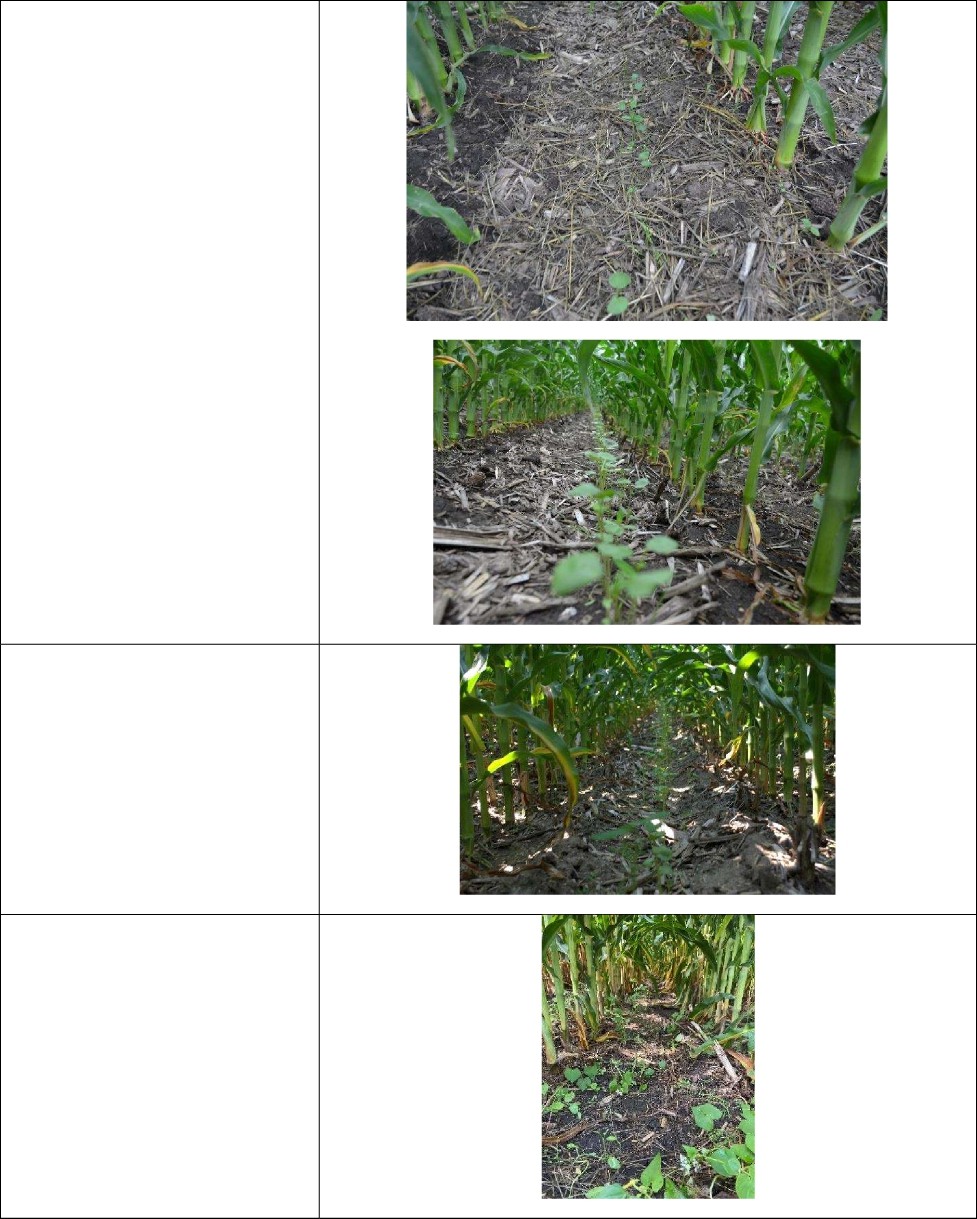
Photos from standard row interseeded cover plots

July 9, 2021

July 23, 2021

August 27, 2021


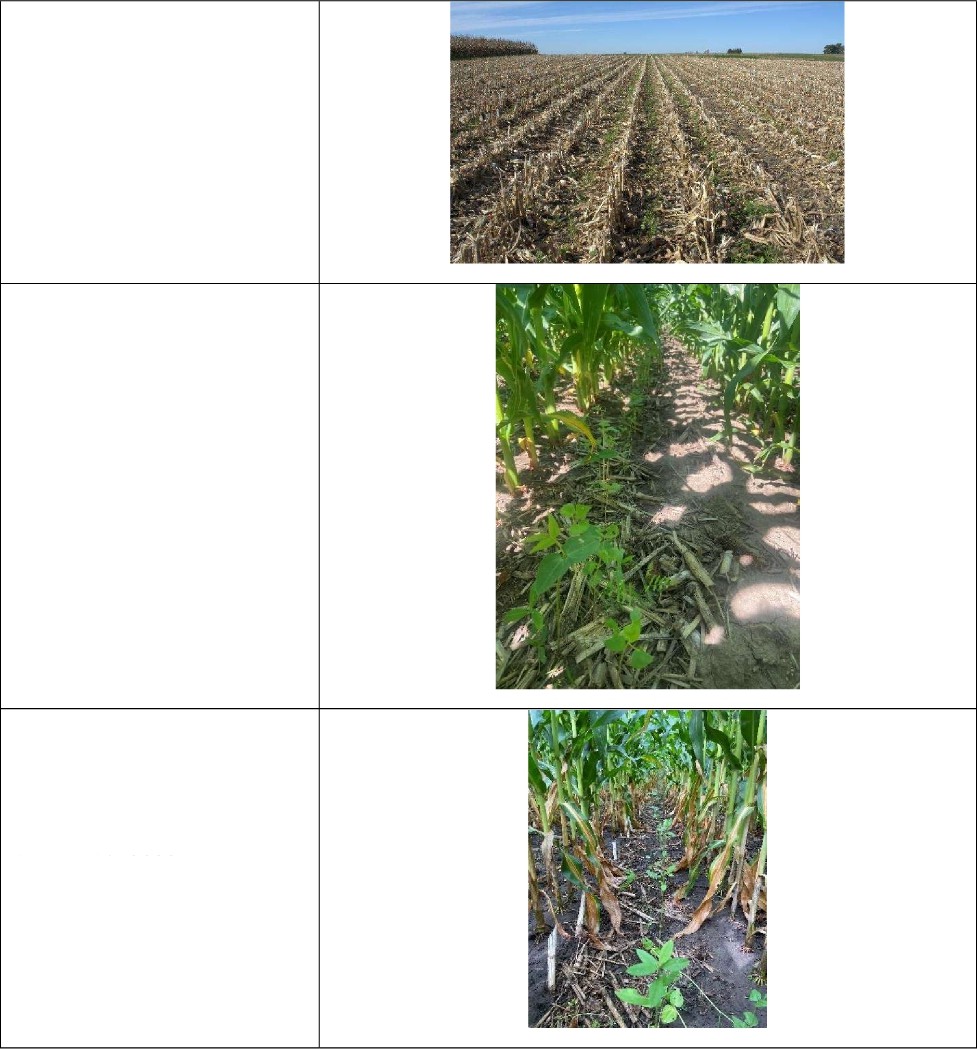


October 15, 2021

July 12, 2022

August 19, 2022


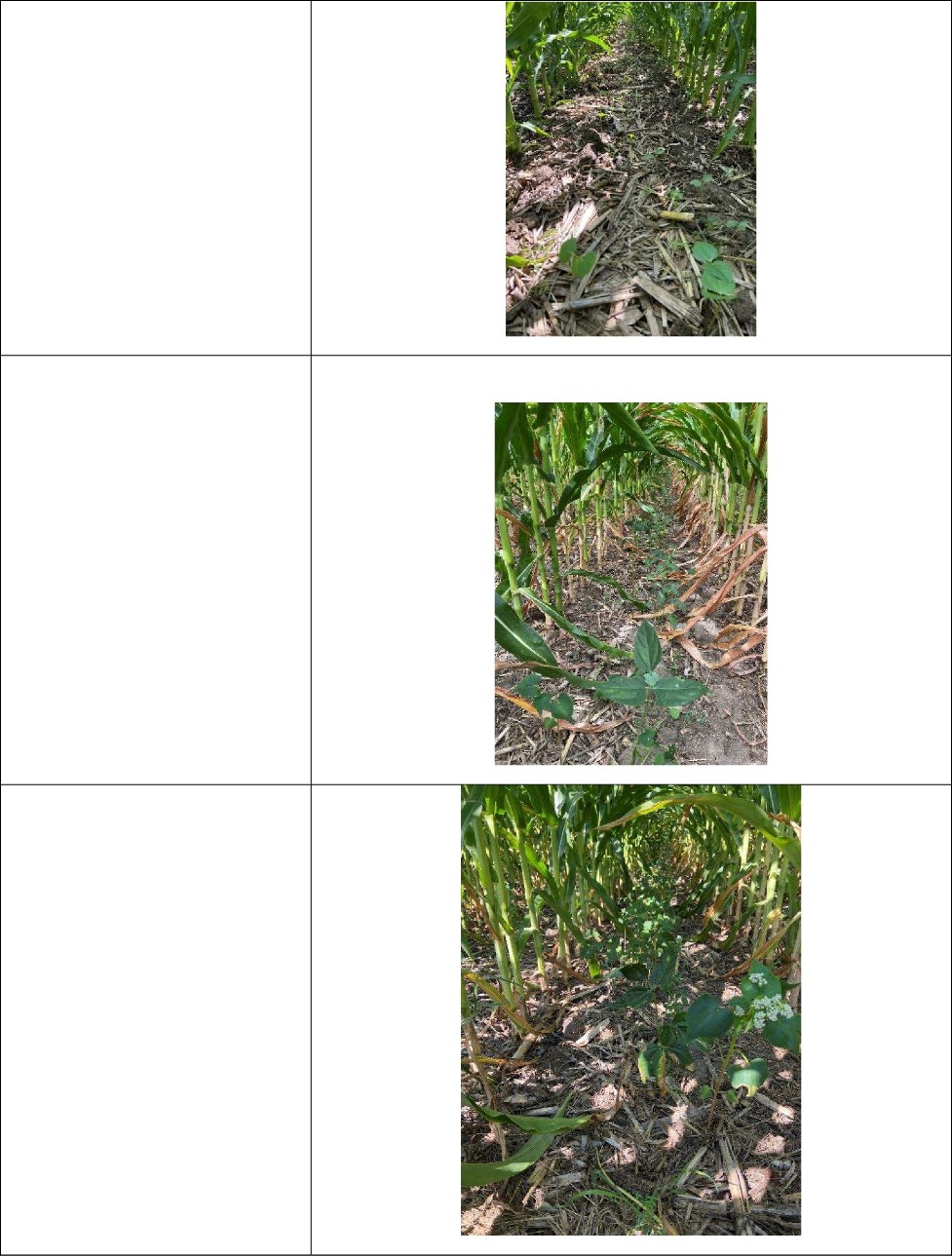


July 6, 2023

August 3, 2023

August 17, 2023


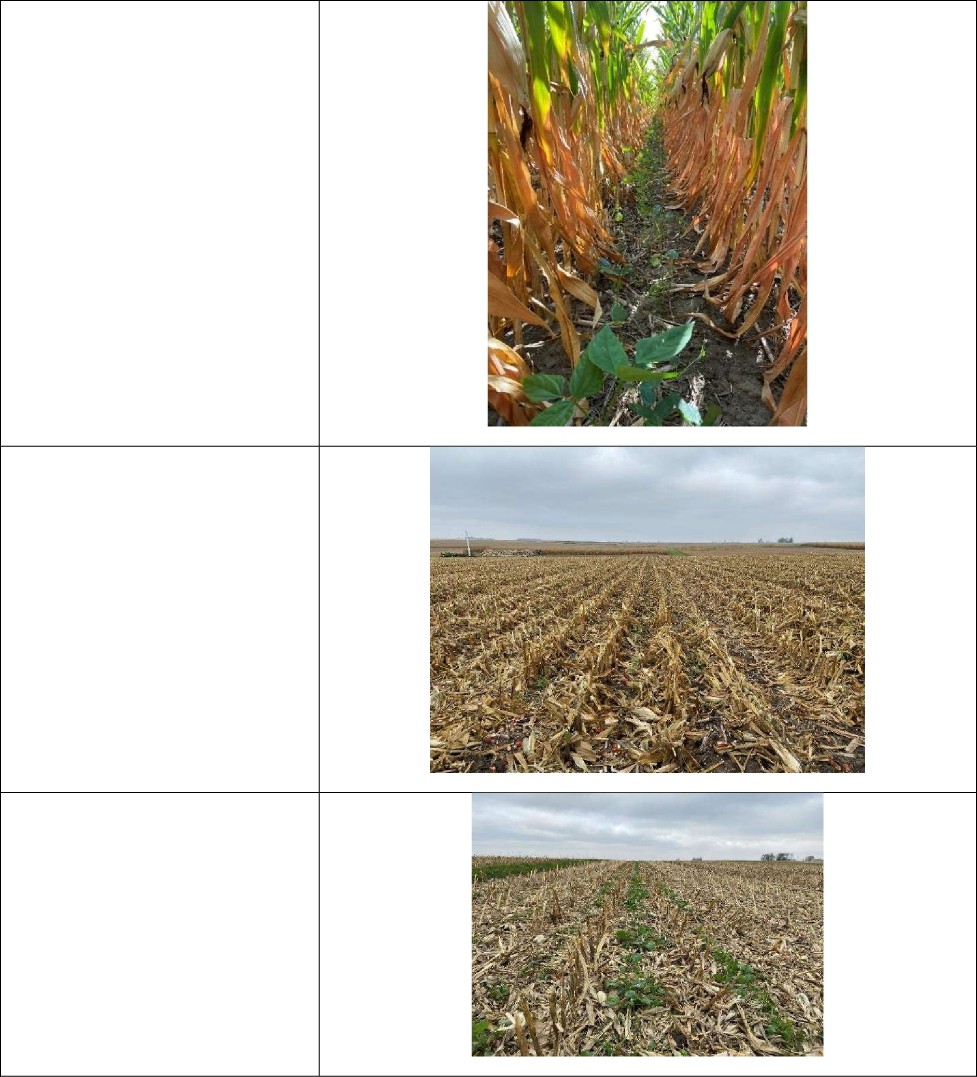


September 1, 2023

September 28, 2023

October 15, 2023

Photos from wide row interseeded cover plots


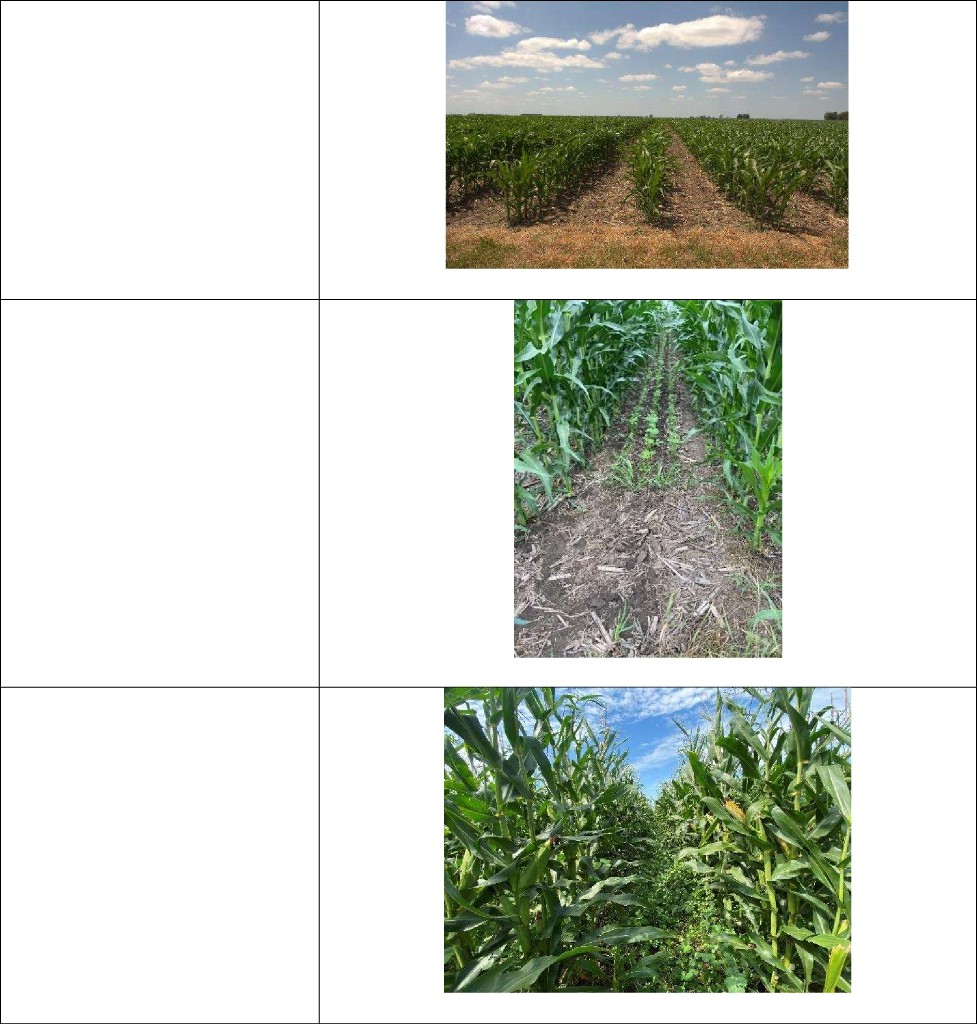


June 23, 2021

July 9, 2021

August 27, 2021


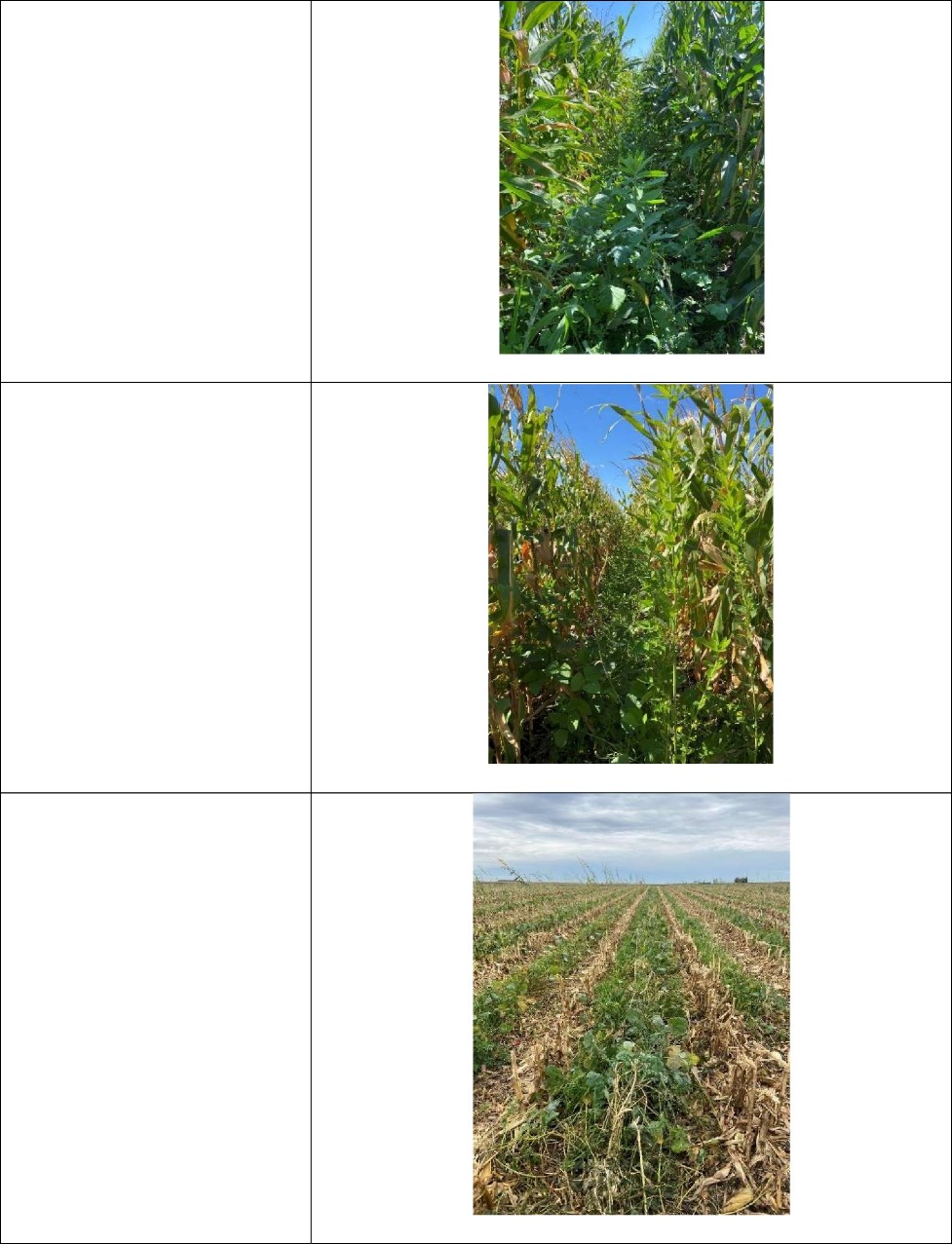


September 10, 2021

September 22, 2021

October 22, 2021


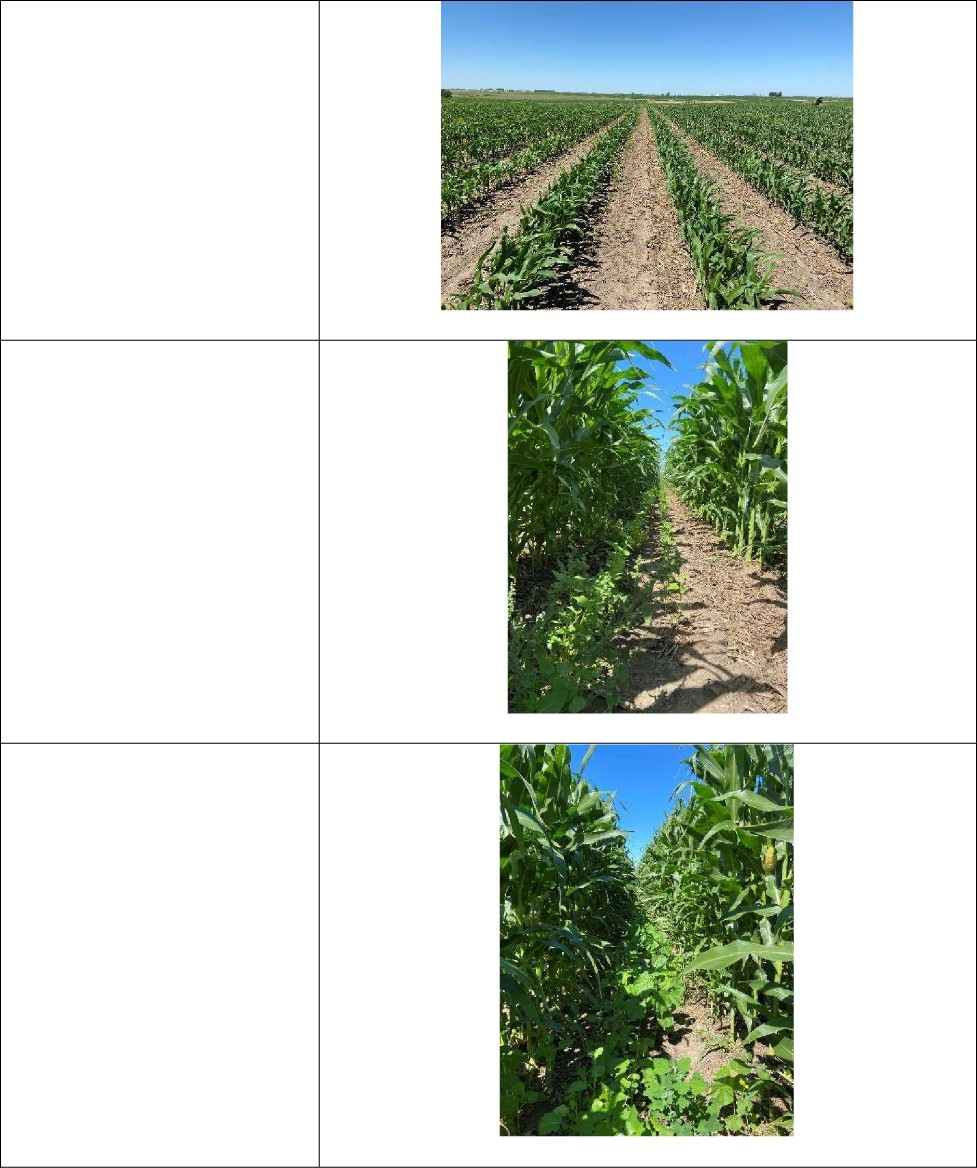


June 22, 2022

July 12, 2022

July 28, 2022


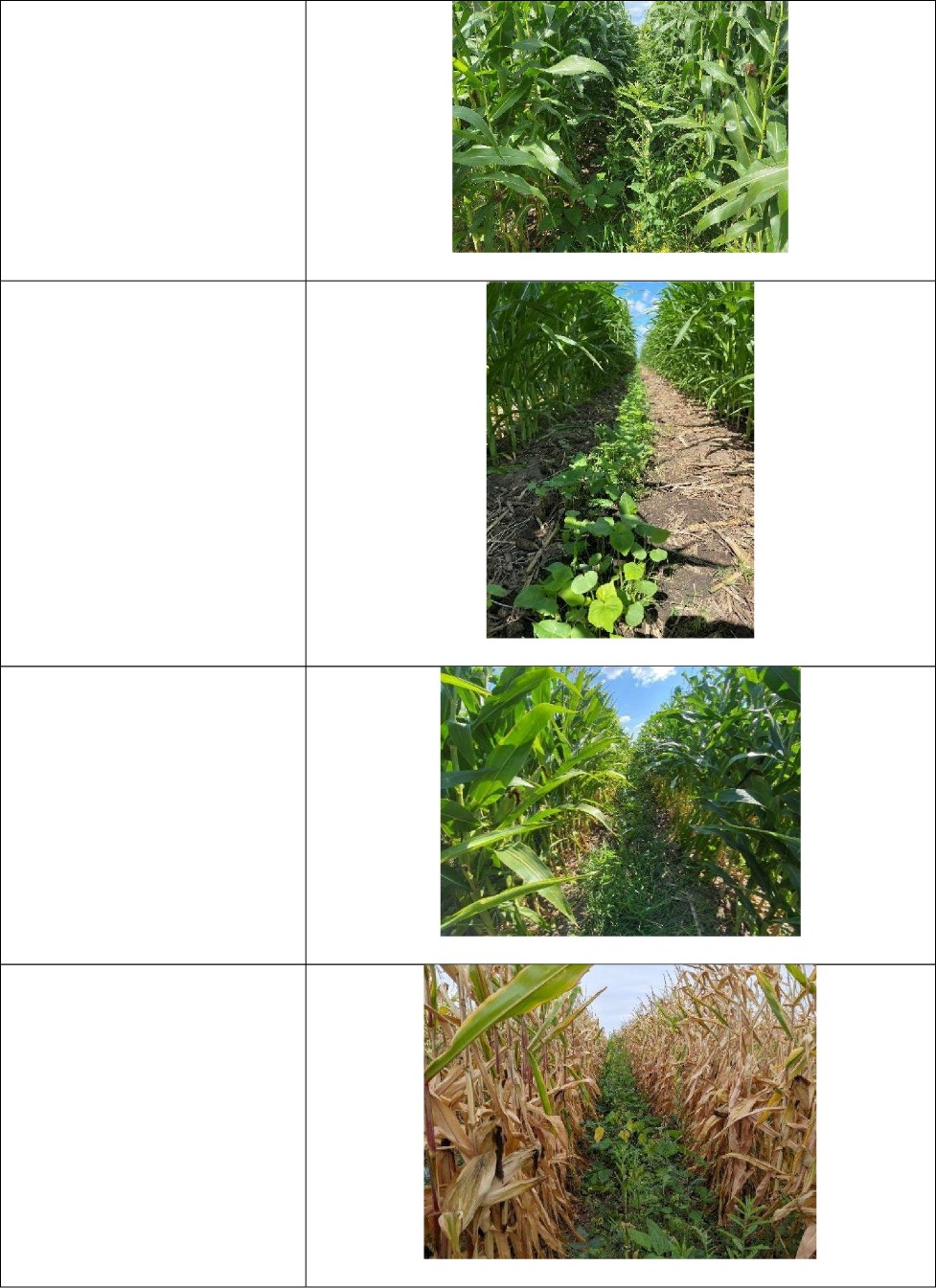


August 19, 2022

July 6, 2023

August 17, 2023

September 15, 2023


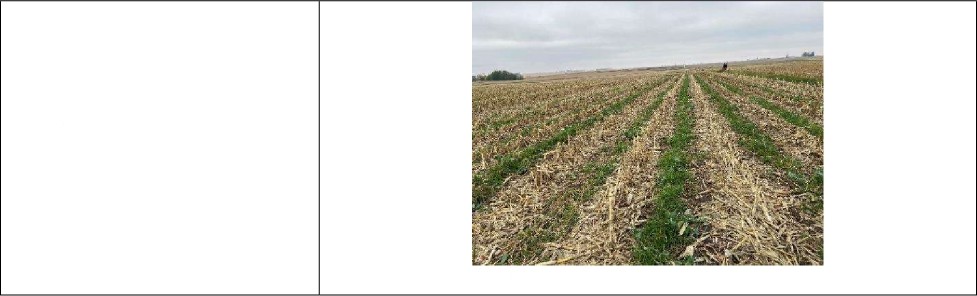


October 15, 2023

Supplemental Table S4. Annual nitrate-nitrogen (NO₃–N) losses (kg N ha⁻¹) from subsurface drainage. Lowercase letters indicate significant differences among treatments within a year. Uppercase letters indicate significant differences among annual averages across years (P ≤ 0.1).

|  | 2021 | 2022 | 2023 | 3-Year Average |
| --- | --- | --- | --- | --- |
|  | | kg N ha-1 |  | |
| No-cover Control | 1.81ab | 24.3 a | 0.039 a | 8.70 |
| Perennial Groundcover | 2.36 a | 18.0 b | 0.42 a | 6.93 |
| Standard Row Interseeded | 1.49 b | 19.6ab | 0.14 a | 7.08 |
| Wide Row Interseeded | 0.94 b | 20.9 ab | 0.12 a | 7.29 |
| Annual Average | 1.65A | 20.7B | 0.18C |  |

¹Statistical letters are not applied to the 3-Year Average column due to the significant Treatment*Year interaction. ²Uppercase letters compare the Annual Average across years. Means with the same uppercase letter are not significantly different.

Supplemental Table S5. Annual dissolved reactive phosphorus (DRP) losses (g P ha⁻¹) from subsurface drainage. Lowercase letters indicate significant differences among treatments within a year. Uppercase letters indicate significant differences among annual averages across years (P ≤ 0.1).

|  | 2021 | 2022 | 2023 | 3-Year Average |
| --- | --- | --- | --- | --- |
|  |  | g P ha-1 |  | |
| No-cover Control | 3.45 a | 7.91 a | 0.56 b | 3.98 |
| Perennial Groundcover | 4.43 a | 7.80 a | 2.26 ab | 4.83 |
| Standard Row Interseeded | 2.58 a | 6.39 a | 1.14 b | 3.36a |
| Wide Row Interseeded | 4.16 a | 12.67 a | 24.71 b | 13.84 |
| Annual Average | 3.65 A | 8.69 B | 0.83 B |  |

¹Statistical letters are not applied to the 3-year average column due to the significant treatment*year interaction. ²Uppercase letters compare the annual average across years. Means with the same uppercase letter are not significantly different.
